# Picture Novelty Influences Response Selection and Inhibition: The Role of the In-Group Bias and Task-Difficulty

**DOI:** 10.1371/journal.pone.0165470

**Published:** 2016-10-27

**Authors:** Artyom Zinchenko, Waich Mahmud, Musrura Mefta Alam, Nadia Kabir, Md. Mamun Al-Amin

**Affiliations:** 1 Department Psychologie, Ludwig-Maximilians-Universität München, Munich, Germany; 2 Max Planck Institute for Human Cognitive and Brain Sciences, Leipzig, Germany; 3 Department of Pharmaceutical Sciences, North South University, Dhaka, Bangladesh; Centre de neuroscience cognitive, FRANCE

## Abstract

The human visual system prioritizes processing of novel information, leading to faster detection of novel stimuli. Novelty facilitates conflict resolution through the enhanced early perceptual processing. However, the role of novel information processing during the conflict-related response selection and inhibition remains unclear. Here, we used a face-gender classification version of the Simon task and manipulated task-difficulty and novelty of task-relevant information. The novel quality of stimuli was made task-irrelevant, and an in-group bias was tightly controlled by manipulation of a gender of picture stimuli. We found that the in-group bias modulated the role of novelty in executive control. Novel *opposite-sex* stimuli facilitated response inhibition only when the task was not demanding. By contrast, novelty enhanced response selection irrespective of the in-group factor when task-difficulty was increased. These findings support the in-group bias mechanism of visual processing, in cases when attentional resources are not limited by a demanding task. The results are further discussed along the lines of the attentional load theory and neural mechanisms of response-inhibition and locomotor activity. In conclusion, our data showed that processing of novel information may enhance executive control through facilitated response selection and inhibition.

## Introduction

Flexible behavioral control is an ability to track and respond to salient changes in a dynamic environment. Indeed, humans rapidly detect and evaluate novel stimuli, irrespective of its task-relevance [1–4]. Processing of novelty seems to have a certain priority in the brain; novel information attracts attention and elicits an orienting reflex [1, 2]. In line with this hypothesis, evidence suggests that novelty facilitates early visual processing and cognitive control [3, 4]. For instance, in a modified Stroop task, Krebs and colleagues [3] showed that novel information attenuates semantic interference, speeds up conflict processing, and enhances visual perception [4].

By contrasts, the role of novel information in *response selection and inhibition* is less clear. Recent evidence suggests that cognitive system employs partially dissociable control mechanisms to resolve conflicts at the early visual processing and response selection stages [5, 6]. In the Stroop task, conflicts are mainly resolved by enhanced processing of the task-relevant information during the early visual processing [7, 8]. Alternatively, in the Simon task, conflicts are resolved by inhibition of the task-irrelevant information during the later response selection stage [9, 10]. Previous studies reported motor slowing after observing an unexpected novel event [11]. Participants were instructed to verbally report one of the two target letters presented on the screen. Prior to the target, a short sound was delivered to participants via the headphones. The results of the study showed greater motor slowing after novel sounds (20% of all trials) relative to familiar sounds (80%). However, the question remains whether novel task-irrelevant component of the target stimulus would influence response inhibition when presented within trials, relative to when presented prior to the target [11]. Additionally, it is not clear whether the influence of novelty on motor inhibition comes from the perceptual saliency of novel objects relative to the surprise value of a stimulus, as novel stimuli usually compose 1/5 of all trials.

The current study investigated the influence of novelty on the response selection and inhibition during a Simon task. Participants were presented with a male or female picture (face) displayed either to the left or the right of the fixation cross. Participants were asked to make a decision whether they saw a female or male picture by pressing either the right or left-hand button. In the congruent condition, the picture occurred in the *same* relative location as the response, while in the incongruent condition the picture and response were in conflict. Also, half of the pictures were familiarized prior to the experiment yielding novel and familiar pictures (picture novelty), which however was task-irrelevant. Previous studies showed that participants automatically code the direction of perceived gaze, although completely irrelevant to the task [12]. Therefore, all the face stimuli in the current study looked straight ahead to avoid the bias.

Furthermore, there is some evidence for the existence of an in-group bias effect when participants tend to pay more attention to and remember the faces of their gender [13–15]. Therefore, half of the stimuli were pictures of males and the other half were female images. This resulted in a 2 (congruent, incongruent) by 2 (novel, familiar) by 2 (male pictures, female pictures) factorial design with a gender of participants as a between-subject factor. Importantly, in line with previous studies [3], novel and familiar pictures occurred with equal probability to ensure that the effect of stimulus novelty is driven by perceptual saliency itself [16, 17] rather than by the mere surprise value of an event [18].

We hypothesized that novel stimuli would facilitate response selection, similar to facilitation of semantic interference [3]. Alternatively, salient task-irrelevant novel stimuli could have a detrimental effect by drawing attention away from the task-relevant information, similar to other task-irrelevant salient stimuli; for example, negative emotions [19]. Additionally, we hypothesized that the in-group pictures would elicit stronger effects in either facilitating or inhibiting response-selection, due to their high saliency and biological relevance [13].

## Experiment 1

### Materials and Methods

#### Participants and experimental procedure

Forty healthy right-handed individuals (mean age: 21.4 years; range: 19–24 years; 20 males) completed a Simon task. All participants were recruited from the student population of the North South University, Dhaka, Bangladesh. Written informed consent was obtained prior to experiment from all of the participants. The experimental protocol was approved by the ethical committee of the School of Health and Life Sciences, North South University.

The stimuli were composed of pictures taken from the database of adult facial stimuli [20] as well as pictures of Bangladeshi males and females (the database was manually collected at North South University). Prior to the testing session, participants completed a familiarization task, in which half of the picture stimuli (80 pictures) were repeatedly presented in random order (four times each) intermixed with 40 non-repeat pictures that were not used in the Simon task [3]. Each picture was presented for 1500 ms with a variable stimulus-onset asynchrony between 1000 and 2000 ms. To ensure that participants looked at all pictures during the familiarization session, we asked to indicate for each picture presentation whether they had seen the current picture before or not by pressing a button (>95% of pictures were reported as familiar after the fourth presentation). All pictures were presented in the center of a grey screen (visual angle 9 × 6°).

**Fig 1:**
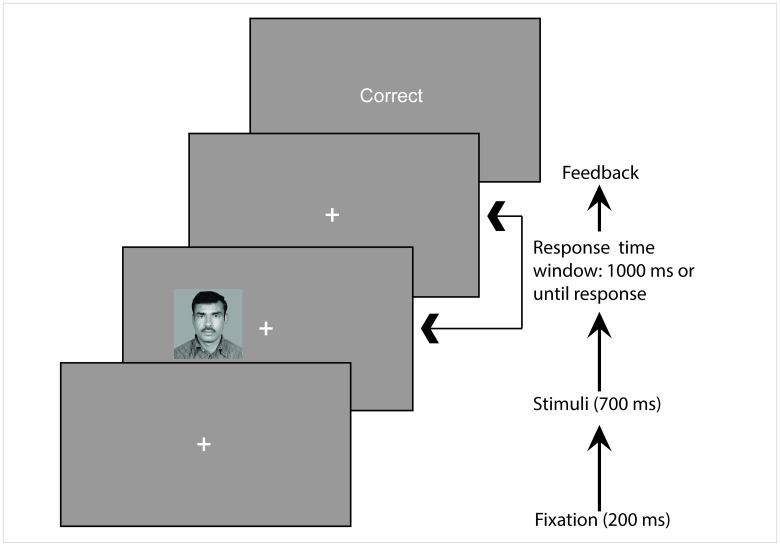
Example of a trial sequence. Each trial started with a fixation cross that was present throughout the whole trial. Afterwards, the target display was briefly presented and participants decided via button press if they saw a male or a female picture. Each trial ended with a visually presented performance feedback.

During the Simon task (Fig 1), the 80 familiarized (50% male) and 80 completely novel (50% male) male and female pictures were displayed once in random order for 700 ms each on either the left (x = 0.02°, y = 0.36°) or the right (x = 0.82°, y = 0.36°) side of the fixation cross (Fig 1). For the stimulus presentation, we used PychoPy stimulus delivery software [21]. In each trial, the side of the stimulus could be either congruent (50%) or incongruent (50%) with the response hand. This resulted in a 2 (congruent, incongruent) by 2 (novel, familiar) by 2 (female, male pictures) factorial design. Gender of participants was a between-subject factor. Participants were instructed to attend to the picture and to decide as quickly as possible whether they saw a male or a female picture by responding with either left or right index finger (counterbalanced across participants). Importantly, the familiarity/novelty manipulation of the pictures was entirely irrelevant to the task. Throughout the experiment, a small fixation cross was visible in the center of the screen and participants were instructed to maintain accurate fixation.

### Data Analysis

The reaction time and error rates were split according to conditions and submitted to the repeated-measures ANOVA using the R software [22]. The RT below and over the 2.5 standard deviations (SD) from the mean were excluded from further analysis, which resulted in < 2% of data excluded from analysis. Importantly, the excluded data did not influence the results of statistical analysis. Finally, only statistically significant main effects and interactions that involve the critical factors of novelty and congruence are reported in the results section.

## Results

### RT

**Fig 2:**
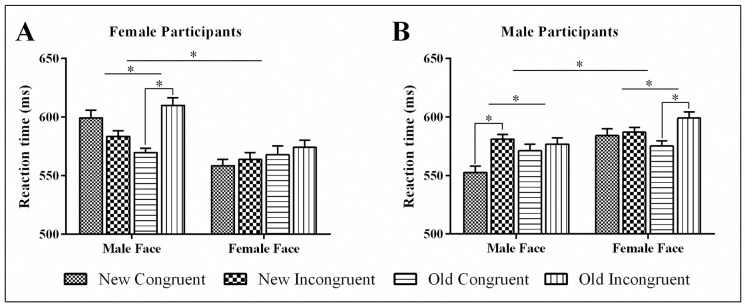
RT data. The figure represents RT data to congruent and incongruent stimuli as a function of novelty, gender of stimuli and gender of participants. The conflict effect is smaller for familiar compared to novel pictures of an opposite gender. For the same-gender pictures, novelty either impeded conflict processing (male participants) or had no effect on conflict processing (female participants).

We observed a four-way interaction of novelty by congruence by gender of the picture by gender of participants (*F*(1, 38) = 26.91, *p* < 0.001, $\eta_{p}^{2}$ = 0.415) and resolved it by participants’ gender (Fig 2). In female participants, novelty influenced cognitive control in the pictures of male (*F*(1, 19) = 23.14, *p* < 0.001, $\eta_{p}^{2}$ = 0.549) but not female stimuli (*F*(1, 19) = 0.005, *p* > 0.9, $\eta_{p}^{2}$ = 0.000). We found that novel male stimuli facilitated cognitive control by reducing the interference effect (*F*(1, 19) = 2.59, *p* > 0.1, $\eta_{p}^{2}$ = 0.120) relative to the familiar male pictures (*F*(1, 19) = 27.67, *p* < 0.001, $\eta_{p}^{2}$ = 0.593).

In male participants, novelty influenced cognitive control in both male pictures (*F*(1, 19) = 7.80, *p* < 0.02, $\eta_{p}^{2}$ = 0.291) and female pictures (*F*(1, 19) = 6.98, *p* < 0.02, $\eta_{p}^{2}$ = 0.269). Novel male pictures inhibited cognitive control by increasing the RT conflict effect (*F*(1, 19) = 15.72, *p* < 0.001, $\eta_{p}^{2}$ = 0.453) relative to the familiar male pictures (*F*(1, 19) = 0.378, *p* > 0.5, $\eta_{p}^{2}$ = 0.019). On the other hand, female novel pictures facilitated cognitive control by reducing the RT conflict effect (*F*(1, 19) = 0.148, *p* > 0.7, $\eta_{p}^{2}$ = 0.008) relative to the familiar female pictures (*F*(1, 19) = 9.54, *p* < 0.01, $\eta_{p}^{2}$ = 0.334).

### Error rate

**Fig 3:**
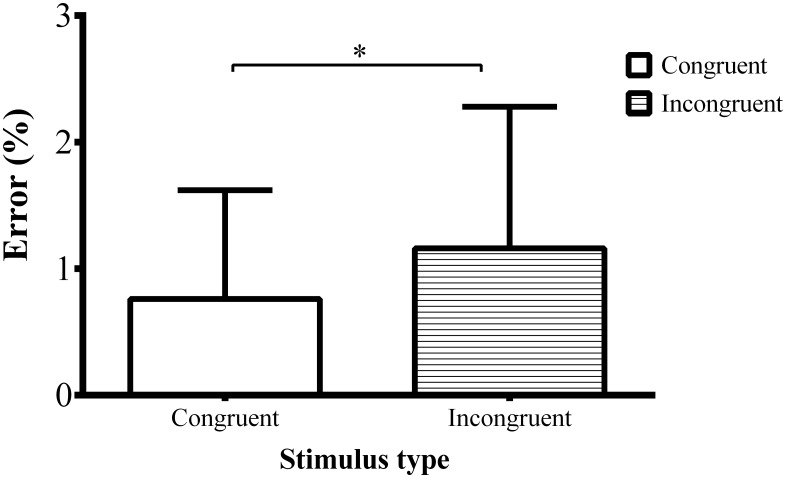
Error rate. The figure represents error rate to congruent and incongruent stimuli. The conflict effect is larger for the own-race stimuli.

Incongruent stimuli produced more errors relative to congruent stimuli (Fig 3; *F*(1, 38) = 14.47, *p* < 0.01, $\eta_{p}^{2}$ = 0.276).

The results showed that novelty facilitated response inhibition in the opposite gender stimuli. However, as the task was easy (error rate ~ 2%), the performance was already at ceiling and the effect of novelty might have not been as pronounced. In other words, due to the ease of the task used in the current set of studies, the cognitive task may not have been demanding enough, and the influence of novelty could also be minimized. Therefore, we conducted Experiment 2 and made the task more demanding to ensure higher error rates.

## Experiment 2

### Participants and experimental procedure

Twenty healthy right-handed individuals (mean age: 22.6 years; range: 19–25 years; 10 males) naïve to the purpose of the study participated in Experiment 2. All participants were recruited from the student population of the North South University, Dhaka, Bangladesh. Written informed consent was obtained before the experiment from all of the participants. The experimental protocol was approved by the ethical committee of the School of Health and Life Sciences, North South University.

The familiarization and experimental procedure were identical to Experiment 1. Additionally, we reduced and randomly varied the presentation duration of stimuli (i.e., 200, 250 and 300 ms).

### Data Analysis

The data analysis procedure was identical to Experiment 1. Only statistically significant main effects and interactions that involve the critical factors of novelty and congruence are reported in the results section.

## Results

### RT

**Fig 4:**
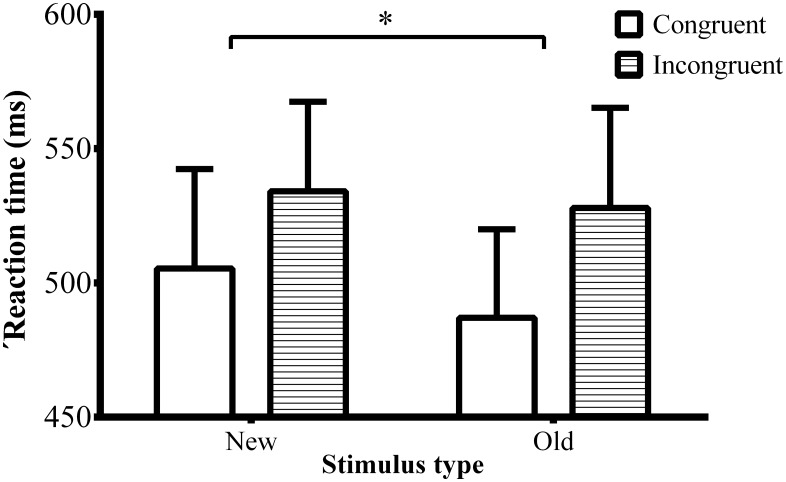
RT data. The figure represents RT data to congruent and incongruent stimuli as a function of novelty. The conflict effect is smaller for familiar compared to novel pictures.

Novel stimuli elicited speeded responses relative to familiar stimuli (Fig 4; F(1, 18) = 13.734, *p* < 0.002, $\eta_{p}^{2}$ = 0.433). Additionally, incongruent stimuli took longer to be processed relative to congruent stimuli (F(1, 18) = 42.309, *p* < 0.001, $\eta_{p}^{2}$ = 0.702). Finally, we observed an interaction of novelty by congruence (F(1, 18) = 5.03, *p* < 0.04, $\eta_{p}^{2}$ = 0.218). Participants were faster to process the incongruence in novel stimuli (F(1, 18) = 17.067, *p* < 0.001, $\eta_{p}^{2}$ = 0.487) relative to familiar stimuli (F(1, 18) = 70.799, *p* < 0.001, $\eta_{p}^{2}$ = 0.797). However, the four-way interaction of novelty by congruence by gender of the picture by gender of participants was not significant anymore (F(1, 18) = 3.51, *p* > 0.05, = 0.163).

### Error rate

**Fig 5:**
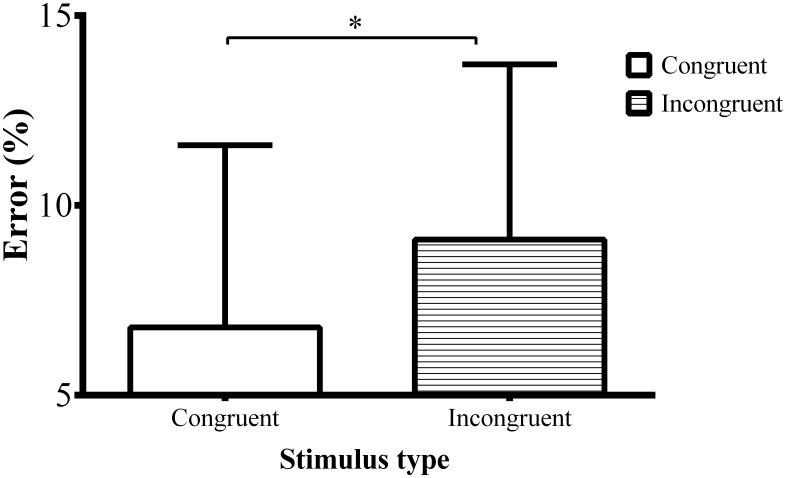
Error rate. The figure represents error rate to congruent and incongruent stimuli. The conflict effect is larger for incongruent stimuli.

Incongruent stimuli resulted in larger number of erroneous responses relative to congruent stimuli (Fig 5; F(1, 18) = 15.999, *p* < 0.001, $\eta_{p}^{2}$ = 0.471).

In summary, Experiment 2 showed that novelty facilitated response selection and inhibition. Importantly, this facilitation seems to be independent of an in-group bias when the task is demanding.

## Discussion

We studied the role of novelty in response selection and inhibition during the cognitive conflict processing. We also determined the role of an in-group bias (gender) in response inhibition and conflict processing as a function of task difficulty. Three main findings emerged: First, participants responded slower and made more errors in incongruent compared to congruent trials. Second, participants responded faster and produced fewer errors when processing pictures of their gender. Third, novel stimuli facilitated response inhibition in the opposite gender stimuli, while novel pictures of the own gender either impeded response inhibition (male participants) or had no influence on response inhibition (female participants). By contrasts, novelty facilitated response inhibition independently of the in-group factor when the task difficulty level was increased.

We replicated previous findings: participants took longer and made more errors to process incongruent than congruent trials [23]. Conflict processing is characterized by increased competition for attentional resources during response selection and inhibition, which prolongs reaction times and increases error rates [24].

We found an own-gender bias effect. We observed that participants were faster and more efficient to process pictures of their gender. Current results are consistent with the previous findings that showed female participants to have biased processing of female than male faces [14]. Notably, previous studies only observed the own-gender bias effect in female but not male participants. For example, female participants can remember more female faces than male faces [25]. In this context, our findings extend the knowledge of the previously observed own-gender bias to male participants (see also [26]). One of the possible explanations for this finding is that evolutionary it makes sense to recognize one’s competitors rather than possible mates [26, 27]. Further, studies have also shown that the same-gender compared to the opposite-gender role models are the most effective in advertisements [28], and the majority of photographs in the fashion magazines are of people of the same gender as the target audience [26]. In contrast to the previous studies, we tested the in-group bias using the cognitive control task, rather than a memory task. Further research may explain the role of the task in the own-gender bias effect.

Additionally, novel compared to familiar stimuli facilitated response inhibition and conflict processing in pictures of an opposite gender and either inhibited (male participants) or had no influence (female participants) on conflict processing in the same-gender pictures. Facilitated attentional allocation for novel compared to familiar pictures during early perceptual processes has been reported in the past [3, 4]. However, the present study has further elucidated the role of novelty in response selection and inhibition, as well as an in-group bias.

Importantly, the in-group bias effect was absent when task-difficulty was increased. As the task in Experiment 1 was not demanding (error rate ~ 2%), attentional resources could be additionally allocated elsewhere and, thus, bias the role of novelty in response selection and inhibition. For instance, studies reported enhanced neural responses to salient emotional compared to neutral stimuli [29–31]. However, such biased processing of emotions was diminished when the corresponding task difficulty was increased. In other words, scarce attentional resources may be preferentially allocated to salient, although task-irrelevant, information when the task is not demanding. However, as the task-difficulty is increased (error rate ~ 10%), attentional resources are redistributed to focus on the task, reducing attention interference by other factors (e.g., stimulus gender).

The present findings are consistent with the attentional load theory [32]. The theory postulates that distracter interference is reduced under the condition of high perceptual load. However, unlike in the attentional load theory, gender of stimuli was not a distractor, but rather a task-irrelevant component of the target. Furthermore, Wessel and Aron [11] showed that unexpected novel events presented before target stimuli may induce motor slowing via a brain mechanism for action-stopping. This motor slowing becomes beneficial when the spatial target location and the corresponding response are in conflict, as people can easily overcome and inhibit prepotent response tendencies. Importantly, unlike the in-group factor in the current study and emotional information in previous studies [31], the effect of novelty remained when the task-difficulty was increased. This finding might suggest that emotion and novelty have a differential mechanism of impact on executive control.

Furthermore, it was also shown that the neural response to reward is not modulated by task-difficulty [33]. There seems to be a link between processing of novelty and reward; reward was shown to facilitate conflict processing in different versions of the Stroop task [3, 34]. For instance, Krebs and colleagues (37) showed that color-naming performance in the classical Stroop task was enhanced on trials with potential-reward versus those without, whereas incongruent reward-related information in a task-irrelevant dimension can impede task performance. The neural basis of the reward-related enhancement of cognitive control may rely on dopaminergic pathways that are known to be involved in both reward processing [35–38] and conflict processing [39, 40]. Interestingly, processing of *novel* information was also shown to activate dopaminergic pathways [41, 42]. Therefore, manipulating stimulus’ novelty may support response selection and inhibition through the dopaminergic pathways [3, 4].

Dopaminergic neurons have projections from the ventral tegmental area (VTA) to the nucleus accumbens (NAc). Both VTA and NAc play an important role in the regulation of locomotor activity [43]. Animal studies suggest that the integrity of the VTA and NAc circuits is required for the manifestation of novelty-induced motor activity [44]. There seems to exist a connection between novelty-induces dopamine circuits, VTA and NAc [45]. Consistent with these findings, our results show that novelty can modulate executive control by facilitating response selection and inhibition.

Simon task requires shielding of the response selection from the task-irrelevant information. It is possible that since same-gender pictures have processing advantage [26], *novel* same-gender pictures might attract attention [3], but away from the task-relevant information, and, thus impede conflict processing. On the contrary, this effect is reversed in the opposite-gender stimuli. Studies reported distinct underlying mechanisms or even different neural populations that code male and female faces [46]. Furthermore, infants categorize and process male and female faces differently with an advantage for the female faces [47]. The observed disadvantage with male faces implies a weaker representation of the male category, at least in babies whose primary caregiver is a female and especially during the first year of life [47, 48]. Possibly, this early exposure-related difference in gender processing may result in the gender-specific processing mechanism later in adulthood. However, this early exposure does not explain the opposite-gender pattern observed in the female group. It is also possible, that processing of novel opposite-gender pictures facilitates disengagement rather than engagement of attention from the task-irrelevant components, thus speeding up conflict processing. However, this question is beyond the scope of the current study and further studies are necessary to elucidate this point.

Several points may have potentially impacted our results. Previous studies showed that culture and state anxiety might shape the way people process facial and novel information [49–51]. However, we have only tested students in Bangladesh, which may limit interpretation of our results. Therefore, future studies should control for culture differences and state anxiety to investigate the role of the in-group bias, task-difficulty and novelty in response selection and inhibition.

## Conclusion

Novel information results in a prioritized perceptual processing in the brain: it attracts attention and enhances visual perception. As a consequence, the novelty was shown to increase motivation, elicit exploratory behavior, and promote learning. However, the role of novelty in response selection and inhibition was less clear. Our results show that novelty can either facilitate or inhibit response inhibition, depending on the in-group bias effect. However, when the task is demanding, the influence of the in-group bias is neutralized.

## Supporting Information

S1 Data Data Matrix of Experiment 1. (XLSX)

S2 Data Data Matrix of Experiment 2. (XLSX)
